# Schema modes and social maladjustment: The mediating role of difficulty in emotion regulation

**DOI:** 10.1016/j.heliyon.2024.e40219

**Published:** 2024-11-07

**Authors:** Yasaman Sepidkar, Saeedeh Zenoozian, Farzane Ahmadi

**Affiliations:** a Clinical Psychology, Zanjan University of Medical Sciences, Zanjan, Iran; b Clinical Psychology, Social Determinants of Health Research Center, Zanjan University of Medical Sciences, Zanjan, Iran; c Biostatistics, Zanjan University of Medical Sciences, Zanjan, Iran

**Keywords:** Difficulty in emotion regulation, Schema modes, Social adjustment

## Abstract

**Background:**

Studies show that emotion regulation as a special form of self-regulation and schema modes as the basis of different states of awareness affect the behavior and cognition of individuals, which can also affect social adjustment in the sense of establishing a balance between the needs of oneself and society. The present study was conducted to investigate the relationship between schema modes and social maladjustment with the mediation of difficulty in emotion regulation in students.

**Method:**

The participants were 670 students from a medical university who were selected by convenient sampling. Questionnaires on schema modes, Bell's social adjustment, and difficulty in emotion regulation were administered. Data analyzed by the R package *robmed*.

**Results:**

The results indicated the mediating effect of emotion regulation difficulty on the relationship between schema modes and social maladjustment. Child modes predict 16 %, coping modes 13 %, parent modes 12 %, and healthy adult modes 10 % of the variance of social maladjustment mediated by difficulty in emotion regulation.

## 1 Introduction

The transition from high school to college is usually associated with the transition out of the family, frequently for the first time in the child's life and it is a significant event for the person. They are faced with academic pressure, roommates, financial struggles, homesickness and mental disorders hence, it is necessary to make a new adjustment to the environment. The concept of adjustment at first proposed by Darwin as an adaptation for survival in the material world. A person adjusts their behavior in a constant, multidimensional, and sophisticated process called adjustment to establish appropriate and productive relationships with other people and their surroundings. One sort of adjustment is social adjustment, which is the ability to compromise and adapt a person to the social environment or change it to match individual characteristics and needs [1]. In other words, social adjustment includes efficiency in social interactions and adherence to social norms, values, and regulations to meet desires. Literature shows that more positive relations in the environment lead to better coping strategies and more resilience or capacity to adapt, it is also related to emotional understanding, empathy, and self-concepts in adolescence [2]. In spite of that, Lack of social adjustment means isolation and the inability to communicate with society and people, which hurts motivation and mental health [3]. Suicide was the third leading cause of death among 15–29-year-olds in 2021 (the majority of college students are within this age range). A combination of individual, relationship, and societal factors contribute to thoughts of suicide and attempted suicide. What is evident is that people who attempt suicide experience intense hopelessness, disconnection from others [4], or loneliness and belongingness [5]. Moreover, In different psychological disorders, such as avoidant personality [6], panic disorder [7], post-traumatic stress disorder [8], mood disorders [9] there are significant challenges in social adjustment and appropriate interpersonal functioning. A study shows that depression was prevalent in about 48.30 % and anxiety was prevalent in 50 % of students [10] unlike this, mutual friendship in adolescence predicts less social anxiety and loneliness [11].

Emotions are complex reactions to events that reflect a change in the individual's welfare, that feel pleasant or unpleasant and that change how people think and behave [12]. Emotion regulation is the use of behavioral and cognitive strategies to change the duration or intensity of experiencing an emotion [13] Therefore, emotion regulation is defined as a basic principle in starting, evaluating, and organizing adaptive behavior, preventing negative emotions and maladaptive behaviors [14]. By regulating emotions, people can modulate their experience of pleasure and pain, control their thoughts and behaviors, and consequently, gain greater control over their social relationships and well-being [15]. Social avoidance and social adjustment problems are associated with lower emotional regulation [16] and effects on social performance and social damage [17] also, emotional reactivity and emotion regulation were important predictors of at least two types of conflict and cooperation social behavior [18]. Research on emotions in education has shown how emotions are a part of academic learning, influencing students' motivation, cognitive processes and academic attainment [19]. Emotion dysregulation predicts adolescent anxiety, aggression, and eating pathology [20] as well as plays a role in psychological disorders such as borderline personality [21], anxiety symptoms [22], disorders related to drug and alcohol abuse [23], social anxiety [24] and depression [25].

Schema modes are another factor that influence on cognition, thoughts, emotions and acts. They are the predominant emotional states and coping responses triggered by oversensitive situations [26]. Young et al. (2003) said the modes are schemas or schema functions that are adaptive or maladaptive and are currently activated in the individual information processing system. It means the modes are different aspects of the self that reflect how we think, feel and behave at the moment [27]. Schema modes are categorized into four general categories child modes, coping modes, parent modes, and healthy adult mode as mentioned in Table 1 [26]. A type of social maladjustment is seen in people with borderline personality disorder with emotional instability and unstable relationships and in antisocial people with a lack of empathy and people abuse, which can be attributed to the high activity of Detached Protector, Punitive Parent, Abandoned/Abused Child, Angry Child and Bully/Attack mode [28]. Also, people with personality disorder experience deficits in emotional processes (e.g., lower behavioral inhibition and resistance to emotion-related impulses), particularly in interpersonal contexts [29]. A study indicated there was a significant relationship between schema modes and emotion regulation [30], and another research showed every mode is related to some emotional dysregulation, and in schema therapy to improve emotion regulation, negative emotions are identified and allowed to be experienced and managed with the help of other modes. In other words, schema therapy treats severe emotional dysregulation using the therapy alliance and empirical techniques focused on emotion [31]. Significant studies indicate the role of modes in maladjustment and various psychological problems, including personality disorders [26], social anxiety disorder [32], obsessive-compulsive disorder [33], and depression [34] Also, Fassbinder and Arntz by presenting a case, state that the techniques used in schema therapy are effective for social inhibition and avoidance, which are the characteristics of third-category personality disorders [35]. Studies show the relationship between emotion dysregulation with social maladjustment, inappropriate social performance [16,36], and psychiatric disorders [37]; moreover, the results show the influence of modes, schemas, and coping styles on psychological disorder [38,39], and interpersonal and emotional functions [40].

University life is when individuals can recognize themselves and their abilities, demonstrate their autonomy, move away from family members, and emphasize peer relations. This developmental process is not the same for all individuals. Certain individuals engage in this process in a healthier and more self-confident manner by establishing healthy relationships with peers and preparing academic and career plans. In contrast, for others, university life may become a developmental crisis, leading to the emergence of physical and mental illnesses as well as adaptation problems, which may cause academic failures and incomplete university education [41]. we tended to investigate the lack of data about the connection of schema modes and emotion regulation on social maladjustment in the non-clinical population of students because the literature mostly focused on the clinical population and interventions to cure the disorders. Students play a significant role in societies and paying attention to mental health and influencing factors and their adaptations is vital. By predicting modes' positive and negative effects on social adaptation, it is possible to estimate the probability of disorders and prepare appropriate treatment and preventive programs to protect them from huge challenges. There is evidence that EMS predict 50 % and 54 % of variance in symptoms of anxiety and depression, respectively. Hence, we extended the literature by pursuing the under aims and hypothesis. First, we hypothesized that social maladjustment is influenced directly and indirectly by the maladaptive modes, predicting higher social non-adaptation, and on the contrary, the healthy modes (happy child and healthy adult) foresee fewer problems in social interaction. Secondly, emotion dysregulation correlated with lower social adaptation and higher activity of maladaptive schema modes. In the end, as a main goal, investigated the mediation effect of emotion regulation difficulty on the relationship between schema modes and social maladjustment.

**Table 1:** Schema modes overview.

Schema modes	Description
**Child modes**	
Vulnerable child	Feelings of helplessness and hopelessness, fear of abandonment
Angry child	Use anger as the first tool to deal with perceived unmet needs or emotional Threats
Enraged child	Intense feelings of anger, being out of control
Impulsive child	Low frustration tolerance and inability to delay gratification
Undisciplined child	Lacking the discipline to achieve necessary or desired goals
**Coping Modes**	
Compliant Surrender	Submissive, self-deprecating feelings and acts, passive permission for others to mistreat him/her to cope with frustration
Detached Protector	Psychologically withdrawn, feelings of emptiness and emotional numbing in order to cope with the pain resulting from experiencing vulnerability
Detached Self-Soother	Compulsive engagement in activities in which s/he feels soothed or distracted from the painful emotions
Self-Aggrandizer	Competitive, grandiose, and abusive behaviors to obtain own needs/desires, low levels of empathy, cravings for admiration by others
Bully and Attack	Manipulative and sadistic behaviors to overcompensate for potential mistreatment, strategically harmful acts toward others
**Parent modes**	
Demanding Parent	Internalized, parents who continually push to meet unrealistic standards, to become ‘perfect' at the expense of being spontaneous and expressing feelings
Punitive Parent	Internalized parents who are criticizing, punishing, and unforgiving, which in turn leads to self-criticism and self-destructive behaviors
**Health modes**	
Happy child	Experiences of sufficient love, feelings of connectedness, and satisfaction with Life
Healthy Adult	Ability to maintain appropriate adult functioning, problem-solving, taking responsibility for own actions, making commitments, and pursuing healthy relationships

## 2 Materials and methods

### 2.1 Participant

This is a cross-sectional study. The participants were 670 students (443 girls and 227 boys) of Zanjan University of Medical Science in Iran which was collected by convenience sampling. The age range was 18–38 and the average was 21 years (max: 37 and min: 18). A large part of the sample was female, single, and medical students.

Most of the participants were single and studying medical science.

### 2.2 Procedure

Questionnaires were distributed online on WhatsApp, Telegram, and Instagram groups and social network channels, as well as paper questionnaires in classes and dormitories affiliated with Zanjan University of Medical Sciences. The data collection lasted for three months, from November to December, in 2022 and most of the questionnaires were filled in bachelor's and professional doctorates. The purpose of the research was explained to the participants and if they were satisfied, they participated in the research. Several paper questionnaires that were not fully answered were removed from the data.

### 2.3 Measures

#### 2.3.1 Demographic information

The items of this form collected information about the participants' age, gender, marital status, and education degree.

#### 2.3.2 Schema mode questionnaire

The SMI includes 124 items that measure the modes of four groups: child modes (Vulnerable Child, Angry Child, Enraged Child, Impulsive Child, Undisciplined Child, Happy Child), coping modes (Compliant Surrender, Detached Protector, Detached Self-Soother, Self-Aggrandizer, Bully and Attack), parent modes (Punitive Parent, Demanding Parent), and healthy adult mode. It is scored on a 6-point Likert scale ranging from ‘never to ‘always’. A higher SMI score represents more presence of the (maladaptive) modes. Lobbestael et al. (2010) showed a good internal consistency of the subscales (Cronbach's alpha ranged from 0.76 to 0.96) [42]. Cronbach's alpha of this test was reported as 0.70 in the Persian-Iranian version [43]. In this study, the overall Cronbach's alpha was 0.96, and for the subscales was from 0.95 to 0.64.

#### 2.3.3 Bell's social adjustment questionnaire

Bell developed this scale in 1961. It consists of five parts: social, emotional, employment, health, and at-home adjustment. The exam consists of thirty-two questions. A mark is awarded for a correct answer on yes, no, and I don't know. Questions “2, 8, 9, 13, 14, 17, 19, 21, 23, 26″ are scored in reverse. A high score indicates isolation and distance from social relationships. Bell reported the reliability of social adjustment with Cronbach's alpha method of 0.67 [44]. In the Persian language version, Rezaei et al. (2020) reported Cronbach's alpha 0.88 [45], and in the present study Cronbach's alpha was 0.85.

#### 2.3.4 Difficulties in emotion regulation scale

This questionnaire includes 36 items and 6 subscales of non-acceptance of emotional responses, difficulty in performing purposeful behavior, difficulty in impulse control, lack of emotional awareness, limited access to emotion regulation strategies, and lack of emotional clarity. The response is on a 5-point Likert scale from 1 (very rarely) to 5 (almost always). A higher score in each of the subscales and the whole scale means more difficulty in emotion regulation. Questions “1, 2, 6, 7, 8, 10, 17, 20, 22, 24, 34″ are scored in reverse. Gratz and Roemer (2004) reported the overall internal reliability of the questionnaire as 0.93 and for each sub-scale as 0.85, 0.89, 0.86, 0.80, 0.88, and 0.84 [46]. In the Iranian version, Karami et al. (2016) reported Cronbach's alpha of this scale as 0.87 [47]. In this study, the total Cronbach's alpha was calculated 0.85.

### 2.4 Data analysis

**Fig. 1:**
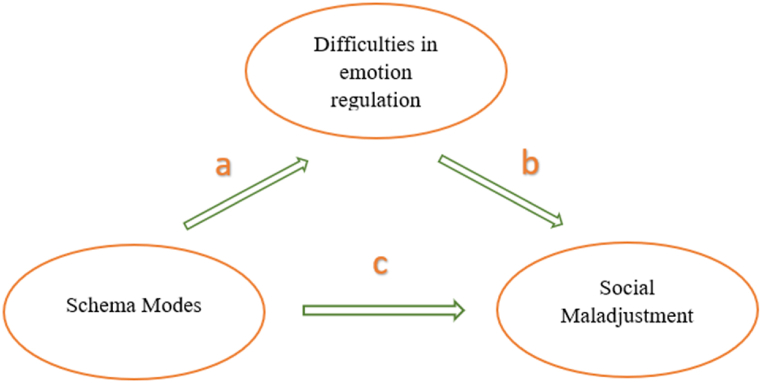
The mediating role of difficulty in emotion regulation in the relationship between modes and social maladjustment.

Determining the mediating role of emotion regulation difficulty in the relationship between schema modes and social maladjustment was measured through mediation analysis with the fit_mediation function in the R package robmed [48], because of the non-normality of dependent variables. This package uses the robust bootstrap method. Fig. 1 conceptually shows the mediating role of difficulty in emotion regulation in the relationship between schema modes and social maladjustment. Due to the schema modes being divided into four groups, the results of each group have been reported separately, we ran the parallel multiple mediator model for each schema modes group.

## 3 Results

### 3.1 Correlational analysis

To investigate the mediating role of emotion regulation difficulty, it is necessary to examine the relationships in Fig. 1. Table 2 shows Spearman's correlation coefficient between social adjustment and schema modes and emotion regulation difficulty. The correlation test indicates a significant relationship between variables.

**Table 2:** Spearman's correlation coefficient of variables.

variables	M	Sd	1	2	3	4	5	6	7	8	9	10	11	12	13	14	15	16
1.social maladjustment	12.9	6.2	–	0.41														
2. difficulty in emotion regulation	2.43	22.13	0.41	–														
3. vulnerable child	2.45	10.99	0.50∗∗	0.67∗∗	–													
4. angry child	2.72	8.69	0.31∗∗	0.6∗∗		–												
5. enraged child	1.81	7.9	0.17∗∗	0.6∗∗			–											
6. impulsive child	2.32	6.6	0.15∗∗	0.56∗∗				–										
7. undiscipline child	2.69	5.06	0.34∗∗	0.54∗∗					–									
8. happy child	3.63	7.25	−0.43∗∗	−0.4∗∗						–								
9. compliant surrender	2.64	5.42	0.36∗∗	0.44∗∗							–							
10. detached protector	2.3	8.14	0.46∗∗	0.6∗∗								–						
11. detached self-soother	3.37	3.68	0.11∗	0.12∗									–					
12. self-aggrandize	3.01	7.51	0.07	0.31∗∗										–				
13. bully & attack	2.74	6.42	0.14∗∗	0.45∗∗											–			
14. punitive parent	1.94	7.99	0.4∗∗	0.66∗∗												–		
15.demanding parent	3.24	8.02	0.23∗∗	0.34∗∗													–	
16. healthy adult	4.18	8.21	−0.41∗∗	−0.54∗∗														–

p < .05. ∗p < .01.∗∗.

### 3.2 Mediation analysis

Table 3 shows the results of the direct, indirect, and total effects of schema modes on social maladjustment with the mediation of difficulty in emotion regulation. Also, the fit index of mediation analysis is reported in Table 4. In the following, the results of each mediation analysis for each schema mode are interpreted. Also, the direct effect of difficulties in emotion regulation on social maladjustment and the direct effect of schema modes on difficulties in emotion regulation are presented in supplementary materials, Tables S1 and S2.

**Table 3:** Direct, indirect, and total effects of different modes on social maladjustment with the mediation of difficulty in emotion regulation.

Mode	Predictor	Direct effect			Indirect effect			Total effect		
		β (SE)	CI 95 %		β	CI 95 %		β (SE)	CI 95 %	
			Lower	Upper		Lower	Upper		Lower	Upper
**Child**	**Vulnerable child**	0.17 (0.04) ∗	0.10	0.25	0.03∗	0.01	0.05	0.20 (1e3)∗	0.13	0.27
	**Angry child**	0.07 (0.04)	−0.01	0.15	0.01∗	1e3	0.03	0.08 (0.05)	-1e4	0.16
	**Enraged child**	−0.11 (0.04) ∗	−0.19	−0.04	0.02 ∗	0.01	0.04	−0.09 (0.02)∗	−0.17	−0.02
	**Impulsive child**	−0.14 (0.05) ∗	−0.24	−0.03	0.02 ∗	0.01	0.04	−0.11 (0.03)∗	−0.22	−0.01
	**Undisciplined child**	0.18 (0.06) ∗	0.06	0.29	0.03∗	0.01	0.05	0.20 (1e3)∗	0.09	0.32
	**Happy child**	−0.23 (0.03) ∗	−0.29	−0.16	−0.03 ∗	−0.5	−0.11	−0.25 (1e3)	−0.31	−0.19
**Coping**	**Compliant Surrender**	0.18 (0.05) ∗	0.08	0.27	0.04 ∗	0.02	0.07	0.21 (0.05)∗	0.11	0.31
	**Detached Protector**	0.25 (0.03) ∗	0.18	0.31	0.08 ∗	0.05	0.11	0.32 (0.03)∗	0.26	0.39
	**Detached Self-Soother**	0.05 (0.06)	−0.07	0.17	0.03 ∗	−0.06	−0.001	0.02 (0.06)	−0.10	0.15
	**Self-Aggrandizer**	−0.07 (0.04)	−0.14	0.01	0.004	−0.01	0.023	−0.07 (0.04)	−0.14	0.01
	**Bully & attack**	−0.13 (0.05) ∗	−0.22	−0.04	0.05∗	0.03	0.09	−0.08 (0.05)	−0.17	0.01
**Parent**	**Punitive parent**	0.08 (0.03)	−0.001	0.15	0.15∗	0.10	0.19	0.22 (0.03)∗	0.16	0.29
	**Demanding parent**	0.11 (0.04) ∗	0.05	0.18	0.01	−0.01	0.03	0.12 (0.04)∗	0.05	0.19
**Healthy adult**	**Healthy adult**	−0.18 (0.30) ∗	−0.24	−0.12	−0.11∗	−0.14	−0.08	−0.29 (0.03)∗	−0.34	−0.23

β: unstandardized regression coefficient **SE**: Standard Error of β**CI**: Confidence Interval ∗: Significant at 0.05 level.

**Table 4:** Fit index for different modes on social maladjustment with the mediation of difficulty in emotion regulation.

Mode	For social maladjustment				For mediator
	R	Adjusted R^2^	F	P-value	R2
**Child**	0.26	0.26	34.60	<0.001	0.16
**Coping**	0.36	0.35	30.88	<0.001	0.13
**Parent**	0.30	0.29	26.32	<0.001	0.12
**Healthy adult**	0.24	0.23	26.01	<0.001	0.10

#### 3.2.1 Child modes

According to the results, vulnerable, enraged, impulsive, undisciplined, and happy child modes predict social maladjustment without mediation, but the relationship between an angry child and social maladjustment was insignificant. In the association between child modes and social maladjustment, difficulty in regulating emotions played a substantial mediating role. In other words, social maladjustment is predicted by child modes through the indirect influence of difficulty in regulating emotions. The regression coefficient of a vulnerable child, an enraged child, an undisciplined child, and an impulsive child was positive, and a happy child was negative. In general, vulnerable child, angry child, impulsive child, undisciplined child, and happy child predict social maladjustment, but the relationship between angry child and social maladjustment was insignificant.

#### 3.2.2 Coping modes

The direct effect of Compliant Surrender, Detached Protector, Bully and Attack modes with social maladjustment was significant, but the effect of Detached Self-Soother and Self-Aggrandizer modes with social maladjustment was not significant. The regression coefficients show that the more active, Compliant Surrender and Detached Protector modes are predictors of maladjustment and social withdrawal But the bully and attack modes have the opposite effect on isolation and maladjustment. Difficulty in emotion regulation mediates the relationship between Compliant Surrender, Detached Protector, Detached Self-Soother, and bully and attack modes with social maladjustment. The regression coefficients of the indirect effect of Compliant Surrender, Detached Protector, Bully, and Attack modeson social maladjustment were positive and the coefficient of Detached Self-Soothermode was negative, which means, a higher score in this mode is associated with less isolation and withdrawal. Based on the total effect, Compliant Surrender, and Detached Protector affect social maladjustment and can predict social isolation.

#### 3.2.3 Parent modes

The demanding parent has a direct effect on social maladjustment, but the effect of the punitive parent on social maladjustment is not significant. Emotion regulation difficulty mediates the relationship between punitive parents and social maladjustment, but the mediation effect on the relationship between demanding parents and social maladjustment is not significant. The regression coefficient of the punitive parent indicates that more activity in this mode predicts more withdrawal and isolation from the social situation. In general, the parent modes can predict social maladjustment and the regression coefficients show a direct relationship, which means more activity of the punitive parent and the demanding parent predict more isolation and social withdrawal.

#### 3.2.4 Healthy adult mode

A healthy adult has a direct and indirect effect on social maladjustment, and its regression coefficient is negative; which means the activity of this mode predicts less isolation and social withdrawal. The total effect of healthy adult mode on social maladjustment is significant and can be predicted with a coefficient of −0.29.

Based on Table 4, the adjusted R^2^ is significant in the four different mediation models, and the child mode mediation model had the largest mediation effect among the four areas of schema modes (R^2^ = 0.16).

## 4 Discussion

As we expected, results show the direct and indirect effect of child modes on social maladjustment. The regression coefficients for a vulnerable, impulsive, angry, enraged and undisciplined child were positive and negative for a happy child; it means, maladaptive modes that mediated difficulties regulating emotions predicted more social disengagement and withdrawal, whereas joyful children that mediated difficulty regulating emotions predicted less maladjustment. Child modes had the most variance (16 %) of indirect effect on social maladjustment indicating greater predictive power of social function; The most common child modes that present distressful are the vulnerable and angry child modes that are characterised by feelings of abandonment, socially unacceptable, undeserving of love, loneliness, frustrated, impatient and resentment, vent their suppressed anger in inappropriate ways or may make demands that alienate others. They developed due to unmet basic needs, childhood neglect or abuse [49]. When a situation threatens to trigger an unpleasant schema, maladaptive coping modes are activated reflecting the coping mode patterns an individual developed in childhood. People are reacting by surrending, avoiding, and Overcompensating. Coping modes often maintain and reinforce underling schemas by preventing individuals from processing and resolving their emotions. In particular, avoidant coping modes function to cut an individual off from the emotional pain associated with their modes [50]. Detached Protector mode withdraws psychologically from the pain of the schemas by emotionally detaching. The individual shuts off all emotions, disconnects from others, rejects their help, and functions almost robotic. Signs and symptoms include depersonalisation, emptiness, boredom, substance abuse, bingeing, self-mutilation, psychosomatic complaints, and blankness. Generally, they do not aware of their and others emotions to get alonge well with people so they experience fewer social adjustment. A compliant Surrender mode faces social challenges by accepting the negative beliefs of a vulnerable child, low self-confidence, self-blaming, rumination, and catastrophizing. Also, bully/attack mode ruins social relations with aggression, insults, and people harm. Based on this, it can be said that students who have deeper schemas and childish mindsets use more incompatible strategies that cause damage to social relationships. Unlike that, Detached self-soother is an avoidant coping mode that demonstrates different outcome which means, predicting better social adaptation indirectly. It is recognized as shutting off emotions by engaging in activities that will somehow soothe, stimulate, or distract a person from feeling. The emotion process hypothesis proposed using this mode as a situation selection guideline (the first emotion regulation strategy). In other words, situation selection is an appropriate and effective strategy in dealing with social situations, which can show the strength of a healthy adult mode in a person.

The outcomes of parent styles and healthy adults mode indicated the mediating effect of emotion regulation difficulty in the relationship between modes and social maladjustment. The regression coefficient was high for the punitive parent (0.15) and it shows this mode noticeably affected social function. A person with an active punitive mode internalizes the voice of parents who criticize and punish the person. They become angry with themselves and feel that they deserve punishment for having or showing normal needs that their parents did not allow them to express. The tone of this mode is harsh, critical, and unforgiving. Signs and symptoms include self-loathing, self-criticism, self-denial, self-mutilation, suicidal fantasies, and self-destructive behavior [49] so, this leads them to the maladaptive strategies such as detached protector, and the consequence of this is withdrawal from the community. These could relate to the not accepting emotion and lack of access to regulatory strategies. Additionally, Healthy adult mode and happy child mode as a person's strength help in using effective regulatory strategies, which cause improved social relations. According to the model, the healthy adult mode predicted 10 % of the variance of social maladjustment by the indirect effect, which confirms the great importance of this mode for a person. As Young et al. (2003) said, this mode has the role of executive function and deals with unmet needs, protection, and discipline of the child and is a healthy part of the personality [27]. Based on the emotion process theory, a healthy mode thinks well, makes good decisions, and uses healthy and logical strategies such as Situation modification, Cognitive change, Response modulation, and expansion of attention to adapt the situation to its needs. Supporting programs to strengthen adaptive and weaken maladaptive modes and emotional management in students helps to prevent mental and psychosomatic disorders and the costs caused by them. They learn to accept themselves, be in the moment, be patient, work, take responsibility, and be committed. The lack of more studies in this area prevented discussion and comparison of the findings. The obtained results were largely in line with the connections seen between schema modes, social maladjustment, and emotion regulation difficulty in the studies mentioned before like Fassbinder and Artz [35], Salgo et al. [30], Yakin et al. [51], Dehghani et al. [52] and Nasrallahi and Pour [53].

The present study had several limitations. First, correlation and mediation analyses prevent conclusions of causation, and self-reporting questionnaires and fatigue caused by the high number of questions could lead to some false outcomes. Second, The study was cross-sectional and we suggest a longitudinal study to understand the temporal relationship between schema mode, emotion regulation difficulties, and social maladjustment also, Identifying each category of modes through interviews and examining them in more detail leads to more reliable results. Third, In this study, social adjustment and emotion regulation were considered as one-dimensional, while they are multi-dimensional, it is better to consider this limitation in future studies with different cultural samples. Conducting a study in other samples would strengthen the conclusion and Future research could incorporate objective measures to corroborate the findings and enhance their validity.

## CRediT authorship contribution statement

**Yasaman Sepidkar:** Writing – original draft, Resources, Investigation, Conceptualization. **Saeedeh Zenoozian:** Writing – review & editing, Supervision, Project administration. **Farzane Ahmadi:** Methodology, Formal analysis, Data curation.

## Ethics and consent

The present study is part of a master's dissertation which was confirmed by Zanjan University of Medical Science with **IR.ZAUMS.REC.1401.144** ethic code. The research participants were over 18 years old and because the goal was not the clinical intervention, written consent was not obtained. The purpose of the research was explained to the participants and they filled out the questionnaires without mentioning their names only in case of personal satisfaction.

## Data availability statement

Data is available by contacting with corresponding author's email.

## Declaration of competing interest

The authors declare that they have no known competing financial interests or personal relationships that could have appeared to influence the work reported in this paper.
