# Supplementary material for: Schema modes and social maladjustment: The mediating role of difficulty in emotion regulation
Source: Heliyon. 2024 Nov 7;10(22):e40219. doi: 10.1016/j.heliyon.2024.e40219 (PMC11616554; doi:10.1016/j.heliyon.2024.e40219)
Supplement: Multimedia component 1 [file mmc1.docx]

**به نام خدا**

دانشجوی گرامی پرسشنامه زیر قسمتی از پایان نامه ارشد روانشناسی بالینی میباشد. ممنون میشم در صورت تمایل به سوالات زیر پاسخ دهید. اطلاعات شما محرمانه میباشد و نامی ذکر نمیشود.

**اطلاعات جمعیت شناختی:**

سن؟ ................ جنسیت؟ ...................

وضعیت تاهل؟ ................. نوع سکونت؟ (خوابگاهی/ بومی)

مقطع تحصیلی؟ کارشناسی/ کارشناسی ارشد/ دکتری تخصصی/ دکتری حرفه ای

**سوالات زیر را باتوجه به پیوستار زیر پاسخ دهید.**

| **5** | **4** | **3** | **2** | **1** | **سوالات** | **ردیف** |
| --- | --- | --- | --- | --- | --- | --- |
|  |  |  |  |  | من در مورد احساساتم صراحت دارم. | 1 |
|  |  |  |  |  | من به چگونگی تجربه احساساتم توجه میکنم. | 2 |
|  |  |  |  |  | من هیجان‌هایم را شدید و خارج از کنترل تجربه میکنم. | 3 |
|  |  |  |  |  | من از احساساتم بی اطلاع هستم. | 4 |
|  |  |  |  |  | من در فهم معنای پشت احساساتم مشکل دارم. | 5 |
|  |  |  |  |  | من به احساساتم توجه میکنم. | 6 |
|  |  |  |  |  | من دقیقا میدانم چه احساسی دارم. | 7 |
|  |  |  |  |  | من به اینکه چه احساسی دارم اهمیت میدهم. | 8 |
|  |  |  |  |  | من در مورد اینکه چه احساسی دارم سردرگم هستم. | 9 |
|  |  |  |  |  | وقتی ناراحتم، به احساساتم اعتنا میکنم. | 10 |
|  |  |  |  |  | وقتی ناراحتم، ازدست خودم عصبانی میشوم که چنین احساسی دارم. | 11 |
|  |  |  |  |  | وقتی ناراحتم، شرم زده میشوم که چنین احساسی دارم. | 12 |
|  |  |  |  |  | وقتی ناراحتم، در انجام کارها مشکل پیدا میکنم. | 13 |
|  |  |  |  |  | وقتی ناراحتم، غیرقابل کنترل میشوم. | 14 |
|  |  |  |  |  | وقتی ناراحتم، میدانم مدت‌ها در آن حالت می‌مانم. | 15 |
|  |  |  |  |  | وقتی ناراحتم، میدانم که درنهایت خیلی احساس غمگینی خواهم کرد. | 16 |
|  |  |  |  |  | وقتی ناراحتم، میدانم که احساساتم موجه و مهم هستند. | 17 |
|  |  |  |  |  | وقتی ناراحتم، به سختی میتوانم روی سایر مسائل تمرکز کنم. | 18 |
|  |  |  |  |  | وقتی ناراحتم، احساس میکنم اختیارم دست خودم نیست. | 19 |
|  |  |  |  |  | وقتی ناراحتم، بازهم میتوانم کارهایم را انجام دهم. | 20 |
|  |  |  |  |  | وقتی ناراحتم، از خودم خجالت میشکم که چنین احساسی دارم. | 21 |
|  |  |  |  |  | وقتی ناراحتم، میدانم که در نهایت راهی پیدا میکنم که احساس بهتری داشته باشم. | 22 |
|  |  |  |  |  | وقتی ناراحتم، احساس میکنم ضعیف هستم. | 23 |
|  |  |  |  |  | وقتی ناراحتم، احساس میکنم میتوانم کنترل رفتارهایم را در دست داشته باشم. | 24 |
|  |  |  |  |  | وقتی ناراحتم، احساس گناه میکنم که چنین احساسی دارم. | 25 |
|  |  |  |  |  | وقتی ناراحتم، تمرکز کردن برایم دشوار است. | 26 |
|  |  |  |  |  | وقتی ناراحتم، در کنترل رفتارهایم دچار مشکل میشوم. | 27 |
|  |  |  |  |  | وقتی ناراحتم، معتقدم کاری وجود ندارد که با انجام آن احساس بهتری داشته باشم | 28 |
|  |  |  |  |  | وقتی ناراحتم، از دست خودم کلافه میشوم که چنین احساسی دارم. | 29 |
|  |  |  |  |  | وقتی ناراحتم، احساس خیلی بدی در مورد خودم پیدا میکنم. | 30 |
|  |  |  |  |  | وقتی ناراحتم، میدانم غرق شدن در ناراحتی تنها کاری است که میتوانم انجام دهم. | 31 |
|  |  |  |  |  | وقتی ناراحتم، کنترل روی رفتارهایم را از دست میدهم. | 32 |
|  |  |  |  |  | وقتی ناراحتم، فکر کردن به هرچیزدیگری برایم دشوار است. | 33 |
|  |  |  |  |  | وقتی ناراحتم، زمانی را اختصاص میدهم که بفهمم واقعا چه احساسی دارم. | 34 |
|  |  |  |  |  | وقتی ناراحتم، خیلی طول میکشد که احساس بهتری پیدا کنم. | 35 |
|  |  |  |  |  | وقتی ناراحتم، هیجان‌هایم را طاقت‌فرسا احساس میکنم. | 36 |

**لطفا سوالات زیر را بادقت پاسخ دهید و نزدیک‌ترین گزینه‌ای که توصفی از شما است علامت بزنید.**

**1................2..................3...................4................5..................6**

هیچوقت به‌ندرت گاهی اوقات اغلب اوقات مدام همیشه

| **ردیف** | **عبارات** | هیچ‌وقت(1) | به ندرت(2) | گاهی(3) | اغلب(4) | مدام(5) | همیشه(6) |
| --- | --- | --- | --- | --- | --- | --- | --- |
| 1 | دیگران باید به من احترام بگذارند و در عین حال حق ندارند به من امر و نهی کنند. |  |  |  |  |  |  |
| 2 | احساس می کنم دیگران مرا قبول دارند و می پذیرند. |  |  |  |  |  |  |
| 3 | خودم را لایق لذت نمی دانم. |  |  |  |  |  |  |
| 4 | احساس می کنم که اساسا آدم بی کفایت، نالایق و بی ارزشی هستم. |  |  |  |  |  |  |
| 5 | برای تنبیه خودم، تکانه های آسیب زایی دارم. (مثل جراحت وارد کردن به خودم) |  |  |  |  |  |  |
| 6 | احساس سرگشتگی می کنم. |  |  |  |  |  |  |
| 7 | برای من سخت است که شخصیت واقعی ام را نشان بدهم. |  |  |  |  |  |  |
| 8 | سخت تلاش می کنم برای جلوگیری از تعارض، جر و بحث یا طرد شدن، مردم دار باشم. |  |  |  |  |  |  |
| 9 | نمی توانم خودم را ببخشم. |  |  |  |  |  |  |
| 10 | ذست به کارهایی می زنم تا محور توجه دیگران قرار بگیرم. |  |  |  |  |  |  |
| 11 | وقتی دیگران به خواسته های من بی توجهی می کنند، عصبی می شوم. |  |  |  |  |  |  |
| 12 | در کنترل تکانه هایم مشکل دارم. |  |  |  |  |  |  |
| 13 | اگر به هدفم نرسم، به راحتی ناکام می شوم و دست از کار میکشم. |  |  |  |  |  |  |
| 14 | خشم و غیظ شدیدی دارم. |  |  |  |  |  |  |
| 15 | تکانشی رفتار میکنم یا سریع هیجان هایم را بروز می دهم به نحوی که موجب آزردگی خاطر دیگران یا آشفتگی خاطر خودم میشوم. |  |  |  |  |  |  |
| 16 | اگر اتفاقات بدی رخ می دهد، به دلیل عیب و ایرادهای من است. |  |  |  |  |  |  |
| 17 | احساس آرامش و راحتی می کنم. |  |  |  |  |  |  |
| 18 | خودم را طبق میل دیگران تغییر می دهم تا مرا دوست داشته باشند یا تاییدم کنند. |  |  |  |  |  |  |
| 19 | احساس می کنم به دیگران تعلق خاطر دارم. |  |  |  |  |  |  |
| 20 | وقتی مشکلی برایم پیش بیاید، سخت تلاش می کنم تا خودم آن را حل و فصل کنم. |  |  |  |  |  |  |
| 21 | برای انجام کارهای روزمره یا خسته کننده، نظم و پشتکار ندارم. |  |  |  |  |  |  |
| 22 | اگر با دیگران نجنگم، از من سوء استفاده می کنند یا مرا نادیده می گیرند. |  |  |  |  |  |  |
| 23 | مجبورم از اطرافیانم مراقبت کنم. |  |  |  |  |  |  |
| 24 | اگر به دیگران اجازه بدهید که به شما زور بگویند یا شما را مسخره کنند، بازنده نهایی شما هستید. |  |  |  |  |  |  |
| 25 | وقتی از دست دیگران عصبانی هستم، به آنها حمله می کنم. |  |  |  |  |  |  |
| 26 | اگر عصبانی بشوم، اغلب نمی توانم جلوی خودم را بگیرم و قشقرق راه می اندازم. |  |  |  |  |  |  |
| 27 | مهمترین مسئله زندگی من این است که نفر اول باشم (مثل مشهورترین، موفق ترین، سالمترین و قدرتمندترین). |  |  |  |  |  |  |
| 28 | نسبت به خیلی چیزها بی تفاوتم. |  |  |  |  |  |  |
| 29 | می توانم مشکلاتم را عاقلانه حل کنم، بدون اینکه اجازه دهم هیجان هایم مرا از پای در آورند. |  |  |  |  |  |  |
| 30 | چاره اندیشی برای موقعیت ها، کاری عبث و مسخره است. |  |  |  |  |  |  |
| 31 | به هیچ وجه نمی توانم توقعاتم را کم کنم. |  |  |  |  |  |  |
| 32 | حمله، بهترین دفاع است. |  |  |  |  |  |  |
| 33 | نسبت به دیگران، بی تفاوت، نامهربان و سنگدل هستم. |  |  |  |  |  |  |
| 34 | کاملا بی تفاوتم (با خودم، با هیجان هایم و با اطرافیانم هیچگونه ارتباطی ندارم). |  |  |  |  |  |  |
| 35 | من مرید چشم و گوش بسته هیجان هایم هستم. |  |  |  |  |  |  |
| 36 | کارد به استخوانم رسیده است (کاملا مستاصلم). |  |  |  |  |  |  |
| 37 | به دیگران اجازه می دهم که مرا دست کم بگیرند و از من انتقاد کنند. |  |  |  |  |  |  |
| 38 | در روابط اجتماعی به طرف مقابل اجازه می دهم که بر من مسلط شود. |  |  |  |  |  |  |
| 39 | احساس می کنم که از مردم فاصله گرفته ام. |  |  |  |  |  |  |
| 40 | بدون فکر، حرف می زنم و دیگران آزرده خاطر می شوند یا بعدا از این کار شرمنده می شوم. |  |  |  |  |  |  |
| 41 | سخت کار می کنم یا به ورزش می پردازم تا به مشکلات آشفته ساز، فکر نکنم. |  |  |  |  |  |  |
| 42 | از دست کسانی که تلاش میکنند آزادی یا استقلال مرا بگیرند، عصبانی میشوم. |  |  |  |  |  |  |
| 43 | احساس می کنم اصلا وجود ندارم. |  |  |  |  |  |  |
| 44 | من بدون توجه به احساس ها و نیازهای دیگران، هر کاری دلم بخواهد انجام می دهم. |  |  |  |  |  |  |
| 45 | اصلا نمی توانم به خودم استراحت و آرامش بدهم یا تفریح کنم، مگر اینکه همه کارها را مطابق معیارهایم انجام دهم. |  |  |  |  |  |  |
| 46 | وقتی عصبانی هستم هر چیزی که دم دستم باشد، پرت می کنم. |  |  |  |  |  |  |
| 47 | از دست دیگران خونم به جوش آمده است. |  |  |  |  |  |  |
| 48 | احساس می کنم که به دیگران تعلق خاطر دارم. |  |  |  |  |  |  |
| 49 | خشم زیادی درون من نهفته است، به گونه‌ای که باید این خشم را رها کنم. |  |  |  |  |  |  |
| 50 | احساس تنهایی می کنم. |  |  |  |  |  |  |
| 51 | تلاش می کنم در هر کاری بهترین باشم. |  |  |  |  |  |  |
| 52 | دوست دارم برای فرار از احساس هایم دست به فعالیت های آرامش بخش یا هیجان انگیز بزنم (مثل کار کردن، خوردن، خرید کردن یا تماشای تلویزیون). |  |  |  |  |  |  |
| 53 | تساوی اصلا وجود ندارد، بنابراین بهتر است که از دیگران برتر باشید. |  |  |  |  |  |  |
| 54 | وقتی عصبانی هستم، اغلب کنترلم را از دست می دهم و دیگران را تهدید می کنم. |  |  |  |  |  |  |
| 55 | به جای اینکه خودم نیازهایم را بیان کنم به دیگران اجازه می دهم راه خودشان را بروند. |  |  |  |  |  |  |
| 56 | اگر کسی با من موافق نباشد، پس بر علیه من است. |  |  |  |  |  |  |
| 57 | برای اینکه کمتر تحت تاثیر افکار و احساسات آشفته ساز قرار بگیرم، باید همیشه دست به دامن کار شوم. |  |  |  |  |  |  |
| 58 | اگر از دست دیگران عصبانی شوم، آدم بدی هستم. |  |  |  |  |  |  |
| 59 | نمی خواهم با مردم ارتباط برقرار کنم. |  |  |  |  |  |  |
| 60 | این قدر عصبانی ام که ممکن است به کسی آسیب بزنم یا او را بکشم. |  |  |  |  |  |  |
| 61 | احساس می کنم که لیاقت دارم که زندگی باثبات و ایمنی داشته باشم. |  |  |  |  |  |  |
| 62 | می دانم که چه موقع هیجان هایم را نشان بدهم و چه موقع دست به این کار نزنم. |  |  |  |  |  |  |
| 63 | اگر کسی من را تنها بگذارد یا به حال خود رهایم کند، عصبانی می شوم. |  |  |  |  |  |  |
| 64 | احساس می کنم با مردم رابطه ندارم. |  |  |  |  |  |  |
| 65 | خودم را وادار به انجام کارهای ناخوشایند نمی کنم، حتی اگر بدانم انجام چنین کارهایی به نفع من است. |  |  |  |  |  |  |
| 66 | قانون شکنی می کنم و بعدش پشیمان می شوم. |  |  |  |  |  |  |
| 67 | احساس شرمساری می کنم. |  |  |  |  |  |  |
| 68 | به بیشتر مردم اعتماد دارم. |  |  |  |  |  |  |
| 69 | اول عمل می کنم، تازه بعدش به کارم فکر می کنم. |  |  |  |  |  |  |
| 70 | خیلی زود بی حوصله می شوم و علاقه ام را از دست می دهم. |  |  |  |  |  |  |
| 71 | حتی در جمع نیز احساس تنهایی می کنم. |  |  |  |  |  |  |
| 72 | نمی توانم به خودم اجازه بدهم که مثل بقیه مردم، دست به فعالیت هایی لذت بخش بزنم چون آدم خوبی نیستم. |  |  |  |  |  |  |
| 73 | خواسته هایم را بیان می کنم، بدون اینکه آرامش خودم را از دست بدهم. |  |  |  |  |  |  |
| 74 | احساس می کنم در مقایسه با بیشتر مردم، آدم بهتر و خاصی هستم. |  |  |  |  |  |  |
| 75 | نمی خواهم از چیزی مراقبت کنم، این موضوع هیچ اهمیتی برای من ندارد. |  |  |  |  |  |  |
| 76 | وقتی که دیگران به من می گویند که چه احساسی باید داشته باشم یا باید چه رفتاری در پیش بگیرم، عصبانی می شوم. |  |  |  |  |  |  |
| 77 | اگر بر دیگران تسلط پیدا نکنید، آنها بر شما مسلط می شوند. |  |  |  |  |  |  |
| 78 | من بدون توجه به پیامدها، احساس هایم را بازگو می کنم یا دست به اعمال تکانشی می زنم. |  |  |  |  |  |  |
| 79 | دوست دارم دیگران را به دلیل نوع برخوردشان با من، مواخذه کنم. |  |  |  |  |  |  |
| 80 | قادرم از خودم مراقبت کنم. |  |  |  |  |  |  |
| 81 | به دیگران خیلی ایراد می گیرم. |  |  |  |  |  |  |
| 82 | برای رسیدن به اهداف و خواسته هایم، تحت فشار دائمی هستم. |  |  |  |  |  |  |
| 83 | تلاش می کنم که مرتکب اشتباه نشوم، چون در غیر اینصورت خودم را تحقیر می کنم. |  |  |  |  |  |  |
| 84 | مستحق تنبیه شدن هستم. |  |  |  |  |  |  |
| 85 | می توانم تغییر کنم، یاد بگیرم و پیشرفت کنم. |  |  |  |  |  |  |
| 86 | می خواهم به افکار و احساسات آشفته ساز، توجه نکنم. |  |  |  |  |  |  |
| 87 | از دست خودم عصبانی هستم. |  |  |  |  |  |  |
| 88 | هیچ احساسی ندارم. |  |  |  |  |  |  |
| 89 | مجبورم در هر کاری که انجام می دهم، بهترین باشم. |  |  |  |  |  |  |
| 90 | برای دستیابی به معیارهایم، لذت، سلامتی و خوشحالی را فدا کرده ام. |  |  |  |  |  |  |
| 91 | از دیگران توقع زیادی دارم. |  |  |  |  |  |  |
| 92 | اگر عصبانی بشوم، کنترلم را از دست می دهم به گونه ای که به دیگران صدمه می زنم. |  |  |  |  |  |  |
| 93 | آسیب پذیرم. |  |  |  |  |  |  |
| 94 | آدم بدی هستم. |  |  |  |  |  |  |
| 95 | احساس امنیت می کنم. |  |  |  |  |  |  |
| 96 | احساس می کنم که دیگران مرا درک می کنند، به حرف دلم گوش می دهند و تاییدم می کنند. |  |  |  |  |  |  |
| 97 | کنترل تکانه هایم غیرممکن است. |  |  |  |  |  |  |
| 98 | وقتی عصبانی هستم، وسایل دم دستم را می شکنم. |  |  |  |  |  |  |
| 99 | اگر بر دیگران سلطه پیدا کنید، اتفاقی برای شما نمی افتد. |  |  |  |  |  |  |
| 100 | به شیوه ای منفعلانه رفتار می کنم، حتی زمانی که مایل به انجام این سبک و سیاق نیستم. |  |  |  |  |  |  |
| 101 | خشم من غیر قابل کنترل است. |  |  |  |  |  |  |
| 102 | به دیگران زور می گویم و آنها را به باد تمسخر می گیرم. |  |  |  |  |  |  |
| 103 | احساس می کنم دوست دارم افراد را به خاطر کاری که در قبال من انجام داده اند، آزرده خاطر سازم یا به باد انتقاد بگیرم. |  |  |  |  |  |  |
| 104 | چون می دانم چه چیزی "درست" یا "غلط" است، بنابراین سخت در تلاشم تا کار درست را انجام دهم و گرنه از خودم عیب جویی می کنم. |  |  |  |  |  |  |
| 105 | اغلب احساس می کنم در دنیا تنها هستم. |  |  |  |  |  |  |
| 106 | احساس ضعف و درماندگی می کنم. |  |  |  |  |  |  |
| 107 | آدم تنبلی هستم. |  |  |  |  |  |  |
| 108 | میتوانم تمام کارهایی را که افراد مهم زندگی ام انجام می دهند تحمل کنم. |  |  |  |  |  |  |
| 109 | احساس میکنم از من سوء استفاده شده یا با من ناعادلانه رفتار کرده اند. |  |  |  |  |  |  |
| 110 | اگر به انجام کاری تمایل پیدا کنم، حتما آن را انجام می دهم. |  |  |  |  |  |  |
| 111 | احساس می کنم از قلم افتاده ام یا نادیده گرفته شده ام. |  |  |  |  |  |  |
| 112 | دیگران را تحقیر می کنم. |  |  |  |  |  |  |
| 113 | احساس خوشبختی می کنم. |  |  |  |  |  |  |
| 114 | احساس می کنم من نباید مطیع همان قواعدی باشم که دیگران مجبور به رعایت آنها هستند. |  |  |  |  |  |  |
| 115 | زندگی من در حال حاضر پیرامون این مسئله می چرخد که چه کارهایی را انجام دادم و چه کارهایی را باید خوب انجام دهم. |  |  |  |  |  |  |
| 116 | خودم را تنبیه می کنم تا مسئولیت پذیرتر از بیشتر مردم باشم. |  |  |  |  |  |  |
| 117 | وقتی احساس کنم غیرمنصفانه به من ایراد گرفته شده، از من سوء استفاده شده یا با من بدرفتاری می کنند، می توانم حقم را بگیرم. |  |  |  |  |  |  |
| 118 | وقتی اتفاق ناگواری برای من رخ بدهد، لساقت همدردی دیگران را ندارم. |  |  |  |  |  |  |
| 119 | احساس می کنم که هیچکس مرا دوست ندارد. |  |  |  |  |  |  |
| 120 | احساس می کنم در واقع آدم خوبی هستم. |  |  |  |  |  |  |
| 121 | اگر لازم باشد برای دستیابی به ارزش هایم، کارهای تکراری و کسل کننده را انجام می دهم. |  |  |  |  |  |  |
| 122 | احساس شوخ طبعی و خودانگیختگی می کنم. |  |  |  |  |  |  |
| 123 | می توانم چنان عصبانی شوم که فردی را بکشم. |  |  |  |  |  |  |
| 124 | از هویت خودم و کاری که برای شادکامی خودم انجام می دهم، احساس خوبی دارم. |  |  |  |  |  |  |

**ضمن خسته نباشید لطفا به سری آخر سوالات نیز پاسخ دهید.**

| **ردیف** | **سوالات** | **بلی** | **خیر** | **نمیدانم** |
| --- | --- | --- | --- | --- |
| **1** | آیا هنگامی که مجبورید از کسی تقاضای کار کنید، احساس ناراحتی و شرم میکنید؟ |  |  |  |
| **2** | آیا دوست دارید که در جشن‌ها و مهمانی‌های نشاط انگیز شرکت کنید؟ |  |  |  |
| **3** | آیا برایتان مشکل است که با غریبه‌ای، سر صحبت را باز کنید؟ |  |  |  |
| **4** | آیا اگر کسی از شما خواهش کند که بدون آمادگی قبلی درباره موضوعی صحبت کنید، به طور قابل ملاحظه‌ای، دچار آشفتگی میشوید؟ |  |  |  |
| **5** | آیا از فرصت‌های اجتماعی کناره‌گیری کرده‌اید؟ |  |  |  |
| **6** | آیا اگر مجبور باشید، برای ترک محفلی که شامل گروهی از افراد است، اجازه بگیرید، دچار پریشانی می شوید؟ |  |  |  |
| **7** | آیا مایلید به جای آشنایان زیاد، دوستان نزدیک و معدودی داشته باشید؟ |  |  |  |
| **8** | آیا اغلب در مهمانی‌ها مورد توجه قرار میگیرید؟ |  |  |  |
| **9** | آیا به آسانی دوست پیدا میکنید؟ |  |  |  |
| **10** | آیا از وارد شدن به اتاقی که گروهی از افراد با هم به صحبت نشسته‌اند، امتناع میکنید؟ |  |  |  |
| **11** | آیا اغلب برای صحبت کردن در میان جمع از ترس این که مبادا اشتباه کنید یا سخن نادرستی بگویید، دچار تردید میشوید؟ |  |  |  |
| **12** | اگر به یک مجلس سخنرانی، دیر برسید و در صندلی‌های عقب جای خالی نباشد، آیا ترجیح میدهید به جای اینکه صندلی‌های جلو را اشغال کنید، در سالن بایستید ؟ (یا آن جا را ترک کنید؟) |  |  |  |
| **13** | آیا در یک مهمانی یا مجلس سعی میکنید با شخصیت مهم حاضر در جلسه ملاقات کنید؟ |  |  |  |
| **14** | آیا گاهی در امور اجتماعی، نقش رهبر را به عهده میگیرید؟ |  |  |  |
| **15** | آیا در حضور اشخاصی که احترام زیادی برایشان قائلید، اما به خوبی با آنها آشنا نیستید، احساس ناراحتی و شرم میکنید؟ |  |  |  |
| **16** | آیا گاهی مسیرتان را عوض میکنید تا از روبرویی با دیگران بپرهیزید؟ |  |  |  |
| **17** | آیا از رقص دسته جمعی لذت میبرید؟ |  |  |  |
| **18** | آیا برایتان مشکل است که در جمع صحبت کنید؟ |  |  |  |
| **19** | آیا تاکنون برایتان بارها اتفاق افتاده است در برابر جمع یا گروهی حاضر شوید و کاری را انجام دهید؟ |  |  |  |
| **20** | آیا هنگامی که در یک مهمانی شام مهم هستید چیزی لازم داشته باشید، به جای این که خواهش کنید آن را به شما بدهند، ترجیح میدهید بدون آن سر کنید؟ |  |  |  |
| **21** | آیا برایتان آسان است که روابط صمیمانه‌ای با جنس مخالف داشته باشید؟ |  |  |  |
| **22** | آیا شروع صحبت با شخصی که الساعه به او معرفی شده‌اید، برایتان مشکل است؟ |  |  |  |
| **23** | آیا در طرح ریزی و هدایت اعمال افراد به عنوان رئیس جلسه، رهبری گروه و ... تجربه‌ای داشته‌اید؟ |  |  |  |
| **24** | اگر مجبور باشید که برای آغاز بحث و گفتگو در میان جمع، فکر یا عقیده‌ای را ابراز کنید، نسبت به خود دچار شک و تردید میشوید؟ |  |  |  |
| **25** | آیا از کمرویی در زحمت هستید؟ |  |  |  |
| **26** | آیا برایتان آسان است که از دیگران تقاضای کمک کنید؟ |  |  |  |
| **27** | هنگامی که نیاز دارید از شخصی که خیلی خوب با او آشنا نیستید چیزی را درخواست کنید، آیا ترجیح می دهید به جای مراجعه مستقیم به آن فرد، یادداشت یا نامه‌ای برای او بفرستید؟ |  |  |  |
| **28** | آیا برای شما مشکل بوده است که به طور شفاهی در برابر گروهی از مردم گزارش دهید؟ |  |  |  |
| **29** | آیا هنگامی که مجبورید به تنهایی در یک مجلس عمومی وارد شوید در حالی که همه در جای خود نشسته اند،احساس شرم و دستپاچگی میکنید؟ |  |  |  |
| **30** | آیا هرگز داوطلب شده‌اید که یک مهمانی خسته‌کننده را روحیه بدهید؟ |  |  |  |
| **31** | آیا گاهی احساس میکنید که مسئولان در برابر کوشش‌های شما مبنی بر انجام دادن وظیفه‌تان در سطح عالی، حق‌شناسی واقعی ابراز نمیکنند؟ |  |  |  |
| **32** | آیا در مهمانی‌ها، مسئولیت معرفی اشخاص را بر عهده میگیرید؟ |  |  |  |

**سپاس فراوان از همکاری شما دانشجوی عزیز!!**
