# Supplementary material for: Schema modes and social maladjustment: The mediating role of difficulty in emotion regulation
Source: Heliyon. 2024 Nov 7;10(22):e40219. doi: 10.1016/j.heliyon.2024.e40219 (PMC11616554; doi:10.1016/j.heliyon.2024.e40219)
Supplement: Multimedia component 2 [file mmc2.docx]

**Supplementary materials**

**Table S1**: Direct effect of difficulties in emotion regulation on social maladjustment

| **CI 95 %** | | $\boldsymbol{\beta}$ **(SE)** | **Mode** |
| --- | --- | --- | --- |
| **Upper** | **Lower** |  |  |
| 0.07 | 0.02 | 0.04 (0.01) * | **Child** |
| 0.09 | 0.05 | 0.07 (0.01) * | **Coping** |
| 0.11 | 0.05 | 0.08 (0.01) * | **Parent** |
| 0.10 | 0.06 | 0.08 (0.01) * | **Healthy adult** |

$\boldsymbol{\beta}$: unstandardized regression coefficient **SE**: Standard Error of $\boldsymbol{\beta}$ **CI**: Confidence Interval *: Significant at 0.05 level

**Table S2**: Direct effect of schema modes on difficulties in emotion regulation

| **Direct effect** | | | **Component** | **Mode** |
| --- | --- | --- | --- | --- |
| **CI 95 %** | | $\boldsymbol{\beta}$ **(SE)** |  |  |
| **Upper** | **Lower** |  |  |  |
| 0.83 | 0.43 | 0.63 (0.10) * | **Vulnerable child** | **Child** |
| 0.47 | 0.02 | 0.25 (0.12) * | **Angry child** |  |
| 0.66 | 0.21 | 0.43 (0.11) * | **Enraged child** |  |
| 0.81 | 0.20 | 0.50 (0.15) * | **Impulsive child** |  |
| 0.91 | 0.30 | 0.61 (0.15) * | **Undisciplined child** |  |
| -0.45 | -0.81 | -0.63 (0.09) * | **Happy child** |  |
| 0.88 | 0.21 | 0.55 (0.17) * | **Compliant Surrender** | **Coping** |
| 1.31 | 0.89 | 1.10 (0.11) * | **Detached Protector** |  |
| 0.10 | -0.82 | -0.41 (0.21) | **Detached Self-Soother** |  |
| 0.32 | -0.20 | 0.06 (0.13) | **Self-Aggrandizer** |  |
| 1.08 | 0.44 | 0.76 (0.16) * | **Bully & attack** |  |
| 1.94 | 1.58 | 1.75 (0.09) * | **Punitive parent** | **Parent** |
| 0.32 | -0.10 | 0.11 (0.11) | **Demanding parent** |  |
| -1.09 | -1.52 | -1.30 (0.11)* | **Healthy adult** | **Healthy adult** |

$\boldsymbol{\beta}$: unstandardized regression coefficient **SE**: Standard Error of $\boldsymbol{\beta}$ **CI**: Confidence Interval *: Significant at 0.05 level
